# Self-harm in individuals who experience binge eating disorder: A systematic review and meta-analysis

**DOI:** 10.1186/s40337-025-01379-8

**Published:** 2025-09-02

**Authors:** Elana Moore, Caroline Clements, Emily Flattery, Anam Bhutta, Peter Taylor

**Affiliations:** 1 Division of Psychology and Mental Health, School of Health Sciences, Faculty of Biology, Medicine and Health, Manchester Academic Health Sciences Centre, University of Manchester, Manchester, UK; 2 Greater Manchester Mental Health NHS Foundation Trust, Prestwich, UK; 3 Manchester University NHS Foundation Trust, Manchester, UK

**Keywords:** Binge eating disorder, Self-harm, Eating disorders, Systematic review, Meta-analysis

## Abstract

**Background:**

Binge Eating Disorder (BED) and self-harm often co-occur and cause great distress. BED is often poorly understood and under-represented in research, despite high prevalence. It is unclear how self-harm in BED populations compares to rates of self-harm in other eating disorder populations or control groups. A systematic review and meta-analysis were undertaken with the aim of investigating how often self-harm occurs along with BED compared to other eating disorders and control groups.

**Method:**

The protocol for this review was pre-registered (PROSPERO reference: CRD42023466201). Online databases, PsycINFO, MEDLINE and Web of Science, were searched using key terms relating to BED and self-harm, for publications dating up to February 2025. Inclusion criteria were studies that measured BED and self-harm. Titles, abstracts and full texts were screened by independent screeners. Sixteen studies were identified. Meta-analyses were completed to determine the odds of self-harm in BED groups compared to other eating disorders and non-clinical control groups. Risk of bias and publication bias were assessed.

**Results:**

There was no difference in rate of self-harm between people experiencing BED compared to those experiencing Anorexia Nervosa (Odds Ratio [OR] = 0.7, 95% Confidence Interval [CI] = 0.4–1.2) or Other Specified Feeding and Eating Disorders (OR = 0.7, 95% CI = 0.4-1.0). Self-harm was 1.6 times more likely in BED groups compared to non-clinical controls (OR = 1.6, 95% CI = 1.0-2.5), and almost half as likely compared to Bulimia Nervosa groups (OR = 0.6, 95% CI = 0.4–0.8).

**Conclusions:**

BED is associated with a higher prevalence of self-harm compared to non-clinical populations and should be approached similarly to other eating disorders in this regard. Increased awareness of the potential for self-harm in BED groups is vital to ensure interventions for eating disorders integrate self-harm prevention. Further research is required in non-western communities and across sexes and gender identities.

**Supplementary Information:**

The online version contains supplementary material available at 10.1186/s40337-025-01379-8.

## Abstract (plain language summary)

Binge Eating Disorder (BED) is a common but often poorly understood mental health problem. There is some evidence that people experiencing BED often also self-harm. This review investigated how BED and self-harm co-occur, compared to self-harm in other eating disorders. Sixteen studies were identified that investigated BED and self-harm. Self-harm in those experiencing BED did not significantly differ to those who experience Anorexia Nervosa or Other Specified Feeding and Eating Disorders. Self-harm was a lot more common in BED groups than people who do not experience an eating disorder, and a lot less common compared to people who experience Bulimia Nervosa. In conclusion, BED co-occurs with self-harm and should be given similar care as other eating disorders. Most studies identified were based in Western female populations. More research is needed in non-western populations and across sexualities and gender identities. Being more aware of self-harm rates in BED groups helps to improve the care available. It is vital treatments for those who experience BED also consider self-harm prevention.

**Supplementary Information:**

The online version contains supplementary material available at 10.1186/s40337-025-01379-8.

## Background

Eating disorders are a major health concern worldwide with an estimated 1.7% of the world population experiencing an eating disorder in their lifetime, with even higher rates in Western countries [1]. Eating disorders can cause great distress and physical health issues, leading to reduced wellbeing and high economic cost to the individual and wider economy [2]. Binge Eating Disorder (BED) has the highest prevalence of any eating disorder, with an estimated 1.5% of the world population affected [1, 3]. BED is characterised by recurrent episodes of binge eating in the Diagnostic and Statistical Manual of Mental Disorders ([DSM], 4), meaning to eat a large amount of food quickly. The binge is associated with a sense of loss of control, feeling uncomfortably full, distress, shame and disgust. Importantly, BED is not associated with compensatory behaviours, such as fasting, purging or over exercise. Similarly to other eating disorder diagnoses, self-harm is often seen in people who experience BED [5], but it is unknown how self-harm in BED compares to other eating disorders or those without eating disorders.

The impact of BED is significant, with almost all (94%) of those experiencing BED reporting lifelong mental health difficulties such as low mood, substance misuse, anxiety and experiences of trauma [6]. BED has been associated with increased physical health needs, difficulties in work/school, impacts on social lives and reduced quality of life [7]. Despite this, almost half (49%) of those experiencing BED never seek help for their distress, particularly if male or from an ethnic minority group [8]. BED is commonly unrecognised, misdiagnosed and underrepresented in clinical and research settings [3, 9]. Stigma around higher weight or body fat levels linked to binge eating cycles [10, 11] and an expectation a person must be underweight to experience an eating disorder [12] has been suggested as factors that contribute to missed BED diagnoses by clinicians. People of higher weights who experience BED face high levels of stigma in western cultures, through social media and by the weight-loss food industry [13, 14]. Alongside stigma, high levels of trauma and disability in BED groups may lead to diagnostic overshadowing and missed eating-related distress, further biasing researchers and clinicians [10, 13]. Research into the impact of BED is vital to improve understanding and support for those affected.

The evidence base consistently suggests eating disorders and self-harm commonly co-occur [5, 15, 16], and the rates of eating disorders and self-harm co-occurring are rising [17]. Self-harm is defined as any self-injury or self-poisoning, regardless of suicidal intent [18, 19]. It’s estimated 40% of individuals experiencing an eating disorder also self-harm, and 22% attempt suicide [15]. Individuals experiencing any type of eating disorder are almost seven times more likely to self-harm than healthy controls [16].

The systematic review and meta-analysis by Kirkpatrick and colleagues [5] calculated pooled prevalence estimates for non-suicidal self-injury (NSSI) across eating disorder diagnoses. Across diagnoses a prevalence of 35% for lifetime NSSI was reported (79 studies), whereas for BED (*k* = 9) a slightly lower estimate of 21% was calculated. This remains higher than general population estimates of NSSI prevalence [20]. The relationship between BED and self-harm remains unclear, however. Whilst self-harm may result from BED, for example as a response to associated distress, it may also be that a history of self-harm increases the risk of BED, for example if binge eating develops as an alternative to self-harm serving a similar function, such as regulating difficult emotional states [21]. Both BED and self-harm may also arise from common causal factors, accounting for their co-occurrence. Reviewing the extant literature concerning the association between BED and self-harm may help elucidate the nature of any association. Self-harm is a robust risk factor for later suicide [22] associated with premature loss of life [23], further increasing risk in this group alongside the impact of BED. Consequently, even where the causal link between BED and self-harm remains unclear, understanding the relative risk of self-harm (past or current) in those with BED compared to other groups is important in considering the clinical needs and risks in this population.

Several recent systematic reviews and meta-analyses focus on the prevalence of self-harm in eating disorders [5, 15, 16, 24, 25]. However, these reviews do not separately estimate the association for different ED diagnoses [16] or only focused on Anorexia Nervosa (AN) or Bulimia Nervosa (BN; 15). Many of these reviews only compared people experiencing BED to healthy controls [16] or made no comparisons at all [5]. To compare BED groups to other eating disorders is vital to determine the level of clinical need and risk across these populations, which in turn can inform service and intervention development.

This systematic review and meta-analysis aimed to investigate rates of self-harm in people who experience BED, and how this compares to other eating disorder populations, as well as other clinical and non-clinical populations. Studies that included data on the occurrence of self-harm within people with BED and within other comparator groups, allowing for a comparison to made, were included. No specific hypotheses were generated, however. To our knowledge, the current systematic review and meta-analysis is the first to focus on the association of BED and self-harm. The present review focuses specifically on comparing the odds of self-harm in people with BED to other populations. Previous review have reported on the (non-comparative) prevalence of self-harm in BED [5].

## Method

### Preparation of systematic review

The systematic review and meta-analysis were prepared and completed using Preferred Reporting Items for Systematic Reviews and Meta-Analyses (PRISMA) 2020 Guidelines [26]. The protocol for this systematic review and meta-analysis was pre-registered with the International Prospective Register of Systematic Reviews (PROSPERO; registration number CRD42023466201). Two deviations were made. First, meta-analyses were conducted with at least three studies available (rather than five as noted in the protocol). This change was made to make the most use of available data. Second, initially we intended to also look at self-harm related thoughts as well as behaviour, but this plan was later dropped to give the review greater focus.

### Study eligibility

To be eligible for inclusion, studies must have included a measure of BED and a measure of self-harm. BED is characterised by episodes of binge eating without compensatory behaviours, consistent with DSM criteria [4]. Measures of BED could include self-report, clinical interview, outcome measure or diagnosis. Studies that included other types of eating disorders were eligible for inclusion if the study provided data for a BED group separately. Studies were required to specify (or be able to provide this information on request) the presence of a given BED diagnosis, which is not equivalent to binge eating behaviour or symptoms, as binge eating is also present in other eating disorders. Measures of self-harm could include frequency or history. Studies were excluded during screening if they were not written in English (as resources for translation were unavailable), do not use original data (e.g. other reviews) or use only qualitative methodology.

### Search strategy

PsycINFO, MEDLINE and Web of Science electronic databases were searched from February 2025 to earliest available records. No restrictions (e.g. publication date) were used on searches. The databases were selected due to their relevance of psychological and medical research.

The following search terms were used with Boolean operators: (“self-harm*” OR “self harm*” OR “self-injur*” OR “self injur*” OR “self-wound*” OR “self wound*” OR “self-mutilat*” OR “self mutilat*” OR “self-poison*” OR “self poison*” OR “parasuicid*” OR “non-suicid*” OR “non suicid*” OR “NSSI” OR “DSH”) AND (“binge” OR “binge eat*” OR “binge disorder” OR “BED”). The terms must be present in either the title, abstract, subject headings or keywords. Medical Subject Headings (MeSH) terms were used where appropriate (‘Binge Eating Disorder’ and ‘self-injurious behaviour’). Details of the search strategy for each database can be found in Supplementary File 1.

### Screening process

Initial searches were completed by author EM in February 2025. All identified studies were added to reference managing software Endnote [27], and duplicates were removed. Studies were first screened sequentially by title and abstract, excluding any that were not eligible but leaving in studies if this was uncertain. Full texts of remaining studies were then screened for eligibility. Screening was completed in parallel and independently by authors EM (first rater) and EF (second rater). Any studies that had not reached an agreement from initial screening were discussed with the full review team and a conclusion was reached.

The reference lists from relevant systematic reviews and meta-analyses [5, 16, 24, 25] published within the last 10 years were also searched for studies not identified through electronic database searches. The reference lists from each included study were also screened for further potential studies (backwards tracking). Studies that cited each included study were also screened for potential inclusion (forwards tracking).

### Risk of bias

Risk of bias was assessed using an adapted version of the Agency for Research and Healthcare Quality tool [28]. The tool provides a quality rating of ‘yes’, ‘no’, ‘partial’ ‘can’t tell’ or ‘not applicable’ in seven relevant domains. Risk of bias was assessed by authors EM and EF independently. Any disagreements in ratings were resolved through discussion.

### Data extraction

Key characteristics of each study were extracted by author EM into a pre-defined data extraction spreadsheet. These characteristics included population demographics, BED and self-harm measures used, other eating disorders investigated, study design and relevant statistics. Key data for the meta-analysis calculations were extracted by authors EM and EF independently. All discrepancies were discussed until agreement was reached.

Calculations were made to determine N values if only percentages were presented by studies. If data presented was split by demographics (e.g. gender or ethnicity), the demographic groups were collapsed to one group to ensure data was comparable to other studies. If studies reported groups of self-harm frequencies (e.g. the number of participants who self-harm once a week, twice a week, etc.), groups were collapsed to allow the sum of presence or absence of self-harm to ensure data was comparable to other studies. If studies reported multiple sub-diagnoses (e.g. AN restricting type and AN binge/purge type), diagnoses were collapsed into over-arching diagnosis type to ensure meaningful comparison with other studies.

For the purposes of this meta-analysis, a decision was made to include Eating Disorder Not Otherwise Specified (EDNOS) and Other Specified Feeding and Eating Disorder (OSFED) diagnoses as one group. The use of the two diagnoses reflects the change in diagnostic criteria from DSM-4 to DSM-5 [4, 29]. The language used was changed and a more detailed specification was provided, but diagnostic criteria largely remained the same [29]. To separate EDNOS and OSFED diagnoses would not be a meaningful separation and would limit meta-analysis statistical power.

### Data analysis

Analysis was completed in R Version 4.4.2 [31] with the *meta* package [32]. A series of random-effects meta-analyses were used to compare study outcomes (i.e., presence of self-harm) between those experiencing BED and other groups, where *≥* 3 studies were available. Groups with less than three studies were not entered into a meta-analysis as this would add little value with so few studies [33].

Separate meta-analyses were completed for different comparator groups (e.g., control groups or other eating disorder diagnoses). We initially planned to also undertake separate meta-analyses for different time periods (e.g. lifetime, past year, past month), however the large majority of studies provided data on lifetime self-harm only. As such meta-analyses were only undertaken focussing on lifetime self-harm, and individual studies that focused on other time periods are discussed separately.

Self-harm can encompass both suicidal and non-suicidal behaviours. Where a study only reported on one form of self-harm (e.g. NSSI) these data were used in the meta-analyses. Where a study reported data on NSSI and suicide attempt separately, and it was not possible to calculate the overall frequency self-harm (i.e., whether someone had engaged in either NSSI or suicide attempt), further information was requested from authors (provided by authors for one study). If no response was obtained the data for NSSI was used in meta-analyses (this was done for one study). NSSI was the most commonly reported form of self-harm and as such we undertook a post-hoc sensitivity analysis, re-running meta-analyses just using NSSI as the outcome.

A random effects model was used due to expected heterogeneity in the studies in terms of design, location, and measurement. Odds Ratios (Ors) were used as the metric of effect size. The I^2^ statistic [34] was used to determine and interpret the percentage of effect size variability that is not caused by sampling error [35]. For all analyses, raw frequencies were entered directly into R to run analyses. Forest plots were produced to visually present results. Risk of publication bias was assessed using funnel plots for meta-analyses. Only meta-analyses with ten or more studies were assessed using funnel plots as recommended [36]. A p value of < 0.05 was considered significant.

## Results

### Screening

**Fig. 1:**
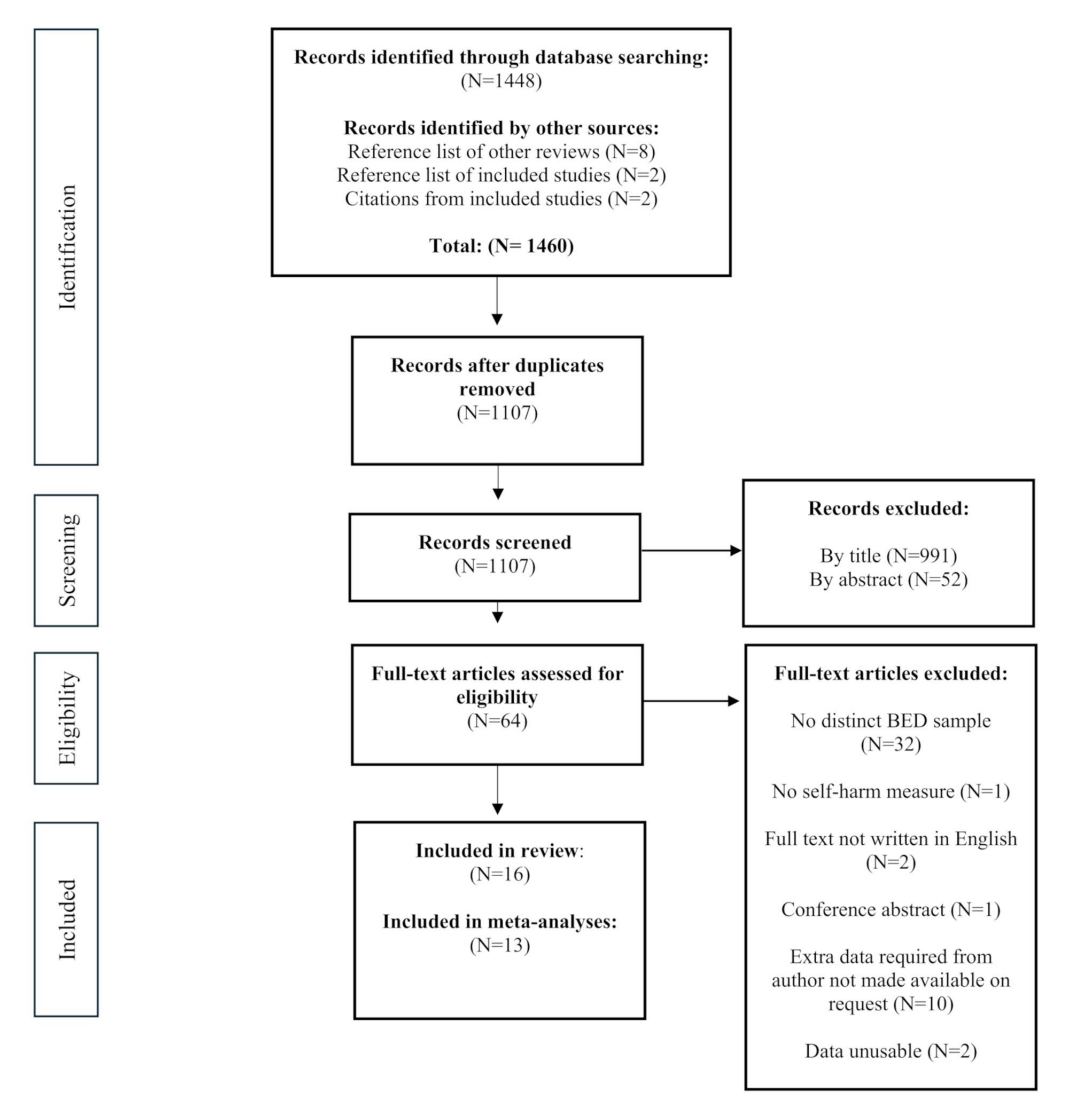
PRISMA Diagram

Figure 1 shows the data identification and screening process (PRISMA diagram). A total of *N* = 1107 studies were identified (after duplicates removed). Following exclusion from title and abstract screening, a total of *N* = 64 full texts were screened. Full text level screening had a 96.9% agreement between independent screeners, before discussion. One paper was initially excluded [37] but subsequently added into the review following discussion. A total of *N* = 16 studies were therefore included.

Studies were excluded at the full text stage of screening for several reasons. A total of 32 studies did not separate BED from binge eating behaviour (i.e., binge eating behaviour may have occurred alongside other eating behaviours and participants no longer met criteria for BED). Where studies discussed BED but did not provide specific data related to BED, authors were contacted for further information (*N* = 11). Of these requests, 1 author provided only partial information which was unable to be used, 1 clarified no participants experienced BED, 1 author reported they no longer had access to the data and 8 authors did not respond.

Two studies met inclusion criteria, but data was unusable in the meta-analysis. One study [38] met inclusion criteria but was excluded due to *N* = 0 within groups (i.e. no presence of self-harm). It is not recommended to use data with *N* = 0, nor recommended to use methods to alter this [36]. A further study [39] met inclusion criteria but did not provide suitable data on a comparison group (i.e. whole sample engaged in self-harm) and was therefore excluded from meta-analyses.

### Study characteristics

Table 1 shows study characteristics. Studies mainly took place in Europe (*k =* 10), alongside North America (*k =* 4), South America (*k =* 1) and Asia (*k =* 1). All studies but two used a cross-sectional design (*k =* 14). The studies focussed on all age adult populations (aged 18 + years old; *k =* 8), adolescent and adult populations combined (13 + years old; *k =* 5) and solely adolescent populations (13–18 years old; *k =* 3). No studies included focused on younger children. A large proportion of the included studies focused on female only samples (*k =* 9). One study also included a non-binary individual (*k =* 1). No other gender identities were reported. No studies reported sex at birth and gender identities separately. Participants were mainly recruited from eating disorder services (*k =* 10), alongside non-clinical populations (*k =* 3), an online eating disorder support group (*k =* 1), university students (*k =* 1), via other research trials (*k =* 1), or as part of a large longitudinal survey (*k* = 1). The number of participants experiencing BED included in the studies ranged from *N* = 7 to *N* = 209. In one longitudinal study the number meeting criteria for BED varied across waves between 30 and 60.

**Table 1:** Key characteristics of included studies

Study	*N* and population*	Recruited from	Design	BED group	Comparison group(s)**	BED measure	Self-harm measure	Self-harm timeframe
Anderson et al., 2023 [43] ^a^ Denmark	*N* = 2509 male and female adolescents	Community	Cross section	*N* = 64	Non-clinical control *N* = 2378	Diagnosis by questionnaire adapted from McKnight Risk Factor Survey (58) and Youth Risk Behaviour Surveillance System (59)	Single-item binary question	Lifetime
Izquierdo et al., 2023 [44] ^c^ USA	*N* = 166 male, female and non-binary adolescents and adults	Eating disorder service	Cross section	*N* = 15	AN *N* = 40 BN *N* = 25 OSFED *N* = 80 ARFID *N* = 6	Diagnosis by Structured Clinical Interview for DSM-IV (41)	Single item binary question	Lifetime
Cella et al., 2022 [45] Italy	*N* = 100 male and female adolescents and adults	Eating disorder service	Cross section	*N* = 39	AN *N* = 46 BN *N* = 15	Diagnosis by Structured Clinical Interview for DSM-IV (42)	Binary outcome by Deliberate Self-Harm Inventory (67)	Lifetime
De Oliveira & Cordás, 2022 [46]^b^ Brazil	*N* = 251 female adolescents	Online eating disorder support group	Cross section	*N* = 65	BN *N* = 62	Diagnosis by Binge Eating Scale (60)	Self-Mutilation Behavioural Scale (68)	Past year
Ahn et al., 2021 [47] ^c^ Korea	*N* = 1355 female adults	Eating disorder service	Cross sectional	*N* = 209	AN *N* = 354 BN *N* = 270 OSFED *N* = 268	Diagnosis by clinical interview and Eating Disorder Inventory – Korean version (61)	Binary outcome by clinical interview	Lifetime
Carlson et al., 2018 [48] ^cd^ Spain	*N* = 220 female adults	Eating disorder service	Cross sectional	*N* = 36	Non-clinical control *N* = 121	Diagnosis by Structured Clinical Interview for DSM-IV (40)	Single item binary question	Lifetime
Smith et al., 2017 [49] USA	*N* = 648 male and female adolescents and adults	Eating disorder and self-harm services	Cross section	*N* = 17	AN *N* = 63 BN *N* = 58 EDNOS *N* = 191	Diagnosis ‘given by psychiatrist’	Single item binary question and frequency per week	Lifetime
Gómez-Expósito et al., 2016 [50] Spain	*N* = 122 female adults	Eating disorder service	Cross section	*N* = 19	AN *N* = 12 BN *N* = 62 EDNOS *N* = 29	Diagnosis by clinical interview and Eating Disorder Inventory (62)	Single item binary question	Lifetime
Micali et al., 2015 [37] UK	*N* ~ 6140 adolescents	Community	Longitudinal	*N* = 30–60 (varies by wave)	People without BED (N varies by wave)	Combination of questions including adapted Youth Risk Behaviour Surveillance Questionnaire (63)	Development and Wellbeing Assessment (DAWBA; 69)	Past year and past month
Claes et al., 2014 [51] ^c^ Belgium	*N* = 99 female adults	Eating disorder service	Cross section	*N* = 22	AN *N* = 36 BN *N* = 41	Diagnosis by clinical interview and Eating Disorder Inventory (62)	Binary outcome by Self- Injurious Questionnaire-Treatment Related (70)	Lifetime
Claes et al., 2013 [52] Spain	*N* = 535 female adolescents and adults	Eating disorder service/ bariatric surgery	Cross section	*N* = 86	AN *N* = 56 BN *N* = 158 EDNOS *N* = 65	Diagnosis by Structured Clinical Interview for DSM-IV (41)	Single item binary question	Lifetime
Iannaccone et al., 2013 [53] Italy	*N* = 65 female adolescents and adults	Eating disorder service	Cross section	*N* = 12	AN *N* = 20 BN *N* = 26	Diagnosis by Eating Disorder Inventory (62)	Single item binary question	Lifetime
Chen et al., 2009 [54] ^e^ USA	*N* = 135 female adults	Participants from a ‘personality disorder’ trial	Cross section	*N* = 7	AN *N* = 9 BN *N* = 8	Diagnosis by Structured Clinical Interview for DSM-IV (41)	Binary outcome by Suicide Attempt Self-Injury Interview (71)	Past year
Fichter et al., 2008 [55] Germany	*N* = 635 male and female adults	Eating disorder service	Longitudinal	*N* = 68	BN *N* = 196	Diagnosis by past records, the Structured Interview for Anorexic and Bulimic Disorders (66), and Eating Disorder Inventory (64)	Single item binary question	Lifetime
Doll et al., 2005 [56] UK	*N* = 1439 male and female adults	University students	Cross section	*N* = 22	AN *N* = 7 BN *N* = 53 Non-clinical control *N* = 1356	Diagnosis by unvalidated questions designed by study	Single item binary question	Lifetime, and present university term
Dohm et al., 2002 [57] ^f^ USA	*N* = 215 female adults	Community	Cross section	*N* = 162	BN *N* = 53	Diagnosis by clinical interview – Eating Disorder Examination (65)	Single item binary question	Lifetime

*Adolescent age group defined as 13–17 years old. Adult age group defined as 18 + years old

**AN = Anorexia Nervosa, BN = Bulimia Nervosa, OSFED = Other Specified Feeding and Eating Disorder, EDNOS = Eating Disorder Not Otherwise Specified, ARFID = Avoidant and Restrictive Feeding and Intake Disorder

^a^ Subthreshold BED has not been included in analysis

^b^Binge eating group used. Most common type of self-harm used as to allow comparison

^c^Anorexia subtypes collapsed to allow comparison

^d^OSFED types collapsed to allow comparison

^e^Self-harm frequency groups collapsed to allow comparison

^f^Purge group used as BN group. Ethnicity groups collapsed to allow comparison

The most common outcome measure for self-harm was presence over the lifetime (*k =* 13). Self-harm in the past year was also used as an outcome measure (*k =* 2) and self-harm over the past academic term (*k =* 1) was also presented. An unvalidated single-item binary question to determine self-harm presence (“yes/no”) was the most common tool used to measure self-harm (*k =* 10). A range of standardised questionnaires was also utilised, but results were presented in binary presence or absence. Some studies presented frequencies of self-harm across a non-specific eating disorder group (*k* = 3). Of those presenting self-harm frequency in specific BED groups (*k* = 2), different timeframes (lifetime vs. past year) were used, meaning frequencies could not be pooled in a further analysis.

Many studies (*k =* 6) reported subgroup data. Subgroups were collapsed to allow meaningful comparison (as reported in Table 1). This included subgroup data that was initially split by demographic (*k =* 1) and split by grouped levels of self-harm frequency (*k =* 1). Diagnostic subtypes of AN (*k* = 4), and OSFED (*k =* 1) were also collapsed. Additionally, in a study that reported frequencies for different self-harm methods, the data for the most common self-harm type was used, as participants may have been counted across multiple self-harm methods (*k =* 1).

### Risk of bias

The outcome of risk of bias assessments can be found in Table 2. A common risk of bias throughout the studies included using unvalidated measures of self-harm. The frequent use of unvalidated single item binary questions increased the risk of bias for over or underestimation of self-harm. A further common risk of bias included a lack of adequate descriptions of participants’ demographic characteristics. Inadequate descriptions of demographic characteristics limit our understanding of the results, particularly in relation to missing ethnicity and socioeconomic data. Furthermore, risk of bias was present by assessors often not being blind to participants’ eating disorder diagnoses (or a lack of clarity on this). A lack of blinding may lead to biased outcomes, as assessors are open to make assumptions or misinterpret data in the light of their knowledge about the participant. There were some risks of bias identified with participant selection, such as online support groups [44] or self-selecting student populations [54], but most used consecutive referrals to eating disorder services. The lowest risk of bias across the studies included using validated measures of BED and minimal missing data.

**Table 2:** Risk of bias assessment

Study	Unbiased cohort selection	Adequate description of cohort	Validated method for assessing clinical status (BED)	Validated measure for assessment outcome variable (self-harm)	Outcome assessments blind to participant status	Adequate follow up period (longitudinal studies only)	Missing data is minimal
Anderson et al., 2023 [43]	Yes	Partially	Yes	No	Yes	NA	Yes
Izquierdo et al., 2023 [44]	No	Yes	Yes	No	Yes	NA	Yes
Cella et al., 2022 [45]	Yes	Partially	Yes	Yes	No	NA	Yes
De Oliveira & Cordás, 2022 [46]	No	Partially	Yes	Yes	Yes	NA	Yes
Ahn et al., 2021 [47]	Yes	Yes	Yes	No	Yes	NA	Yes
Carlson et al., 2018 [48]	Partially	Partially	Yes	No	No	NA	Yes
Smith et al., 2017 [49]	Yes	Yes	Can’t tell	No	Can’t tell	NA	Yes
Gómez-Expósito et al., 2016 [50]	Yes	Partially	Yes	No	Can’t tell	NA	Yes
Micali et al., 2015 [37]	Yes	Yes	Yes	Yes	Yes	Yes	Yes
Claes et al., 2014 [51]	Yes	Partially	Yes	Yes	Yes	NA	Yes
Claes et al., 2013 [52]	Yes	Partially	Yes	No	Yes	NA	Yes
Iannacone et al., 2013 [53]	Yes	Partially	Yes	No	Can’t tell	NA	Yes
Chen et al., 2009 [54]	Yes	Yes	Yes	No	No	NA	Yes
Fichter et al., 2008 [55]	Yes	Partially	Yes	Yes	Can’t tell	Yes	Yes
Doll et al., 2005 [56]	No	Yes	No	No	Yes	NA	Yes
Dohm et al., 2002 [57]	Yes	Yes	Yes	No	No	NA	Yes

### Analyses of self-harm in BED compared to other groups

Table 3 shows the outcomes of the meta-analyses. Four meta-analyses were completed: BED compared to AN, BN, EDNOS/OSFED and a non-clinical control group. All meta-analytic comparisons inspected presence of self-harm over the lifetime. Forest plots were generated to show meta-analysis results visually (Supplementary File II). There were not enough studies to complete meta-analytic calculations for an Avoidant and Restrictive Feeding and Intake Disorder (ARFID) comparison group (*k =* 1), or for outcomes (self-harm presence) for timeframes of one year (*k =* 2) and over an academic term (*k =* 1).

**Table 3:** Results of the meta-analyses

Comparison*	Self-harm timeframe	k	Odds ratio	95% Confidence interval	*p* value	I^2^
BED vs. BN	Lifetime	12	0.60	0.43–0.84	0.003	41.0%
BED vs. AN	Lifetime	10	0.73	0.44–1.22	0.23	53.9%
BED vs. EDNOS/OSFED	Lifetime	6	0.66	0.42–1.04	0.07	39.0%
BED vs. non-clinical control	Lifetime	3	1.58	1.01–2.49	0.05	0.0%

*AN = Anorexia Nervosa, BN = Bulimia Nervosa, EDNOS = Eating Disorder Not Otherwise Specified OSFED = Other Specified Feeding and Eating Disorder

Individuals experiencing BED were significantly more likely (Odds Ratio [OR] = 1.58 95% Confidence Interval [CI] 1.01–2.49) to experience self-harm compared to non-clinical control groups. No heterogeneity was found in this comparison (I^2^ = 0.0%). It is important to note this meta-analysis included only 3 studies, and thus the effect size estimate may not be robust. No funnel plot was completed given the low number of studies included. It was not possible to undertake a sensitivity analysis focused only on NSSI data as only two studies provided this.

A significant difference in the odds of self-harm was found between those experiencing BED compared to BN, with those diagnosed with BED being less likely, *k* = 12, OR = 0.60, CI 0.43–0.84, I^2^ = 41.0%, to experience self-harm compared to those experiencing BN. BED vs. BN funnel plot indicated some asymmetry suggesting some publication bias, which may lead to an inflated effect size in this analysis. Results were similar when the meta-analysis was re-run only for NSSI data, *k* = 8; OR = 0.63, CI 0.42–0.94, I^2^ = 38.8%.

Results showed no significantly different odds of self-harm between BED and AN, *k* = 10, OR = 0.73, CI 0.44–1.22, I^2^ = 53.9%, and BED and EDNOS/OSFED groups, *k* = 6; OR = 0.66, CI 0.42–1.04, I^2^ = 39.0%. BED vs. AN meta-analysis indicated some publication bias due to asymmetry in the funnel plot (Supplementary File III). No funnel plot was completed for BED vs. ENDOS/OSFED meta-analysis given the low number of studies included. Results were similar when the meta-analysis was re-run only for NSSI data: BED vs. EDNOS/OSFED, *k* = 6; OR = 0.63 CI 0.39–1.04, I^2^ = 46.7%; BED vs. AN, *k* = 8; OR = 0.85 CI 0.59–1.22, I^2^ = 39.2%.

### Comparisons not included in a meta-analysis

Several comparisons could not be included in a meta-analysis. Firstly, two studies (*k =* 2; 44, 52) focused on self-harm over the past year (rather than lifetime). These studies were not included as an individual may have engaged in self-harm longer than 1-year ago, but within the lifetime, meaning the timeframes were incomparable. One study [52] recruited participants diagnosed with borderline personality disorder that had taken part in a randomised control trial looking at. The study reported lower rates of past year self-injury in the BED group (71%; *N* = 5) compared to those with AN or BN (75–89%). Although the BED group still experienced more self-injury than those without a comorbid ED 67% (*N* = 74). Likewise for suicide attempts in the past year, the BED group had lower rates (57%; *N* = 4) compared to AN or BN (67% − 100%) but similar to those without an ED (57%). This study utilised very small sample sizes in eating disorder groups and results should be approached cautiously (e.g. *N* = 7 for BED group). Another study [44] recruited through online eating disorder support groups. They found picking at a wound was the most common method of self-harm, with almost half (49%) of participants who experienced BED reported using this method over the past year.

One study investigated self-harm over an academic term [54], this found 4% of those who experienced BED, 14% of those who experienced AN, 2% of those who experienced BN and 2% of non-clinical student controls experienced self-harm in the past academic term. These differences were reported as statistically significant, but it is important to note the very small samples in eating disorder groups (e.g. BED group *N* = 1). This study also reported on suicide attempts specifically (in addition to a general question about harming oneself) but reported no suicide attempts across diagnostic groups having occurred during the term.

One study investigated eating disorder diagnosis ARFID [42] in participants recruited from an eating disorder service. This study found 0% of those identified as experiencing ARFID (*N* = 6) engaged in NSSI whilst one had made a suicide attempt. This result should be interpreted tentatively given such a small sample size.

Two studies investigated frequency of self-harm in BED groups [43, 52]. However, these papers used different measures of frequencies in BED groups, reporting a binary response to ‘5 or more’ [43] and ‘0, 1, 2+’ [52]. Only two studies reported methods of self-harm in BED groups [43, 44]. The most common types of self-harm in BED groups were reported as self-cutting in 66.7% of the BED group [43] and picking at a wound in 49% of the BED group [44]. These studies used different approaches to assess self-harm method and therefore could not be reliably compared.

There were two longitudinal studies. One investigated self-harm and BED over 12 years (*k =* 1; 53). The study recruited participants who were admitted for inpatient treatment and followed up 12-years later using a psychiatric interview to determine the level of improvement in ED symptoms. Bivariate associations were found between self-harm at baseline and ED related outcomes at follow-up, but none of these effects remained significant whilst adjusting for other baseline variables. This study did not control for variation in ED symptom severity at baseline, which is a limitation. A second study used data from a larger-scale longitudinal survey of young people in the UK [37]. BED (assessed over two waves and modelled as a time-varying predictor) was not associated with self-harm occurrence at the following wave. Whilst the overall sample was large (*N* > 4,000) the number with BED was relatively modest, however (*N* = 30–60), which will have affected power.

Three studies provided mixed results regarding lifetime suicide attempts in ED patients and students. In one suicide attempts occurred at a slightly higher prevalence in BN (36%) and AN (42%) groups than BED (11%; 48). Another in students likewise reported attempts were more common in the BN (22%) and AN (14%) groups compared to BED (4%; 54). In contrast the other study reported suicide attempts were more common in the BED group (27%) than BN (12%) or AN group (15%; 42). These studies are all limited by small participant numbers, however.

## Discussion

This systematic review and meta-analysis investigated the co-occurrence of self-harm in people experiencing BED and compared this to the presence of self-harm in other eating disorders and non-clinical samples. This review extended knowledge from recent reviews that identified prevalence of self-harm in people experiencing BED [5, 16, 24, 25]. Results suggested that individuals experiencing BED have 1.6 times the odds of also experiencing self-harm in their lifetime compared to non-clinical controls and did not significantly differ from AN or EDNOS/OSFED. However, the odds of self-harm were less likely in BED groups compared those experiencing BN.

The present review further added to the current knowledge base (e.g., 5, 15, 16), by comparing BED to AN, BN, EDNOS/OSFED and non-clinical control groups. No other reviews to our knowledge investigated these comparisons. These comparisons are particularly important as they indicate that self-harm remains a risk in BED populations, to a similar extent to other eating disorder populations such as AN and EDNOS/OSFED. As the majority of studies focused on lifetime self-harm, and did not explore the timing of events, it remains difficult to elucidate the nature of the relationship between BED and self-harm. For example, it is not clear if self-harm typically precedes or co-occurs with BED. Longitudinal studies were scarce. There was evidence that self-harm was associated with poorer outcomes in BED (though not when adjusting for other factors) and in one study BED was not predictive of later self-harm. There was evidence from two studies that self-harm within the past year was common in BED samples. Self-harm typically serves a function (e.g. regulation of emotions, communication of distress; 21) and so may be a response to distress and impairment arising from BED. It is also possible that binge eating behaviours develop after self-harm in some people as an alternative means of regulating negative emotions, replacing self-harm. There is evidence that binge eating in BED may be triggered by negative affect and could serve an emotion-regulation function, although support for the latter claim has been mixed [72]. The typical age of onset for BED has been reported as the late teens or early twenties [73] which is similar to self-harm [74].

Self-harm in the past is a robust predictor for future self-harm risk [75], and so even if self-harm predates a BED diagnosis this might be indicative of ongoing future risk of self-harm. As self-harm is a strong clinical indicator of later suicide and premature loss of life [22, 23], the findings of this systematic review suggest that clinicians should be mindful of the risk of self-harm within BED populations. It is important that risk in BED groups is not underestimated, and is treated equally to other eating disorders, to ensure support is in place to prevent self-harm and suicide. These findings also further highlight the need for a better understanding of BED, given its high prevalence in comparison to other eating disorders but underrepresentation in research and clinical settings [3, 9].

The risk of self-harm was lower in those with BED compared to BN. This is in line with previous research. A large meta-analysis [5] found higher prevalence of non-suicidal self-injury in BN groups (37.0%) than BED groups (21.2%). The present review furthered this finding by comparing these groups directly, which had not previously been investigated. The difference in self-harm in BED compared to BN groups may be explained by differences in the extent of negative self-referential emotions and emotional regulation difficulties. In comparison to BED groups, BN groups report more extreme eating disorder cognitions in response to ‘feeling fat’ related emotions [76], greater emotional regulation difficulties [77] and high levels of self-hate [78]. Supressing emotions and lack of access to emotional regulation tools predict self-harm [79], and emotional dysregulation mediates the relationship between self-hate and self-harm [80], which may explain why BN groups have higher odds of self-harm. Whilst both BED and BN groups face high levels of stigma, BN groups often face higher levels of blame [14], likely further contributing to high levels of distress. Additionally, according to the acquired capability perspective [81], the exposure to purging (i.e., a high level of physical pain) may weaken the fear of self-harm or suicide, and/or self-harm may weaken the fear of purging, which may further explain the higher odds of self-harm in BN groups compared to BED groups observed in this review. Notably, a large UK based longitudinal survey of young people found BN was significantly associated with greater odds of self-harm at later waves (adjusted OR = 5.72) further supporting the link between BN and self-harm [37].

The studies in this review were often biased to female and western populations. Representation of male or transgender individuals in eating disorder research is often low, despite increasing rates of eating disorders in these groups [82, 83]. Unfortunately, the present review cannot contribute to this under-represented area, given a lack of original studies investigating BED in these populations. Additionally, most studies included took place in Europe and the USA. Other reviews suggest high prevalence of eating disorders in Asian [84] African and indigenous [85], Middle Eastern, Eastern European and Latin American communities [86]. The present review did not find any studies that investigated self-harm and BED in these populations, though this may be explained by excluding non-English language studies. The present review illustrates the disproportionate representation of western communities in eating disorder research. Furthermore, the studies included did not frequently present demographic data such as ethnicity, sexuality, neurodivergence or socioeconomic status, further limiting our understanding and generalisability of the results.

The methods used by identified studies to assess outcomes may also limit our interpretation of the results. Most studies measured presence or absence of BED by an unspecified clinical interview or a Structured Clinical Interview for DSM [40–42]. Previous research has suggested healthcare clinicians have difficulty identifying BED, influenced by barriers in healthcare systems and stigma often linked to higher body weight [9, 11, 12, 14]. This may be reflected in the studies identified in the present review, with most recruiting more participants experiencing BN, despite BED being estimated as the most common eating disorder [1, 3]. Missed diagnoses may be a systemic issue, influenced by the lack of research in BED compared to AN and BN [15, 25]. It is therefore possible many individuals experiencing BED were not accurately captured by the included studies.

Self-harm was commonly measured as a binary variable over the lifetime. This method limits our understanding of the frequency, causes, change over time and nature of the self-harm. The scarcity of longitudinal research further limits our understanding of temporal characteristics of the relationship between BED and self-harm. Studies that consider the timing of self-harm in relation to BED are required to better elucidate their relationship. Some included studies in this review utilised standardised questionnaires, but ultimately binary presence or absence of self-harm was presented as a primary outcome.

This area of research may be at risk of publication bias. As seen in funnel plots for BED vs. AN, and BED vs. BN meta-analyses, there was some indication that amongst smaller studies those with more pronounced effect sizes were more likely to be published. Whilst potentially problematic, it is unlikely this has had a substantial impact on the results given the smaller weighting of these studies within meta-analyses.

The present review excluded studies that were not written in English, meaning otherwise eligible studies from different countries may have been excluded. However, it was noted that only four studies were excluded (over title, abstract and full text screening) for being in a language other than English. It is also recognised that while no limitations were placed upon non-published pieces of work, further efforts could have been made to capture work outside of peer reviewed journals. During the search process, it was noted a significant number of studies investigated binge eating behaviour but not BED (as seen in Fig. 1). As binge eating behaviours feature across other types of eating disorders, these studies were not included. Further research should aim to investigate the association that binge eating behaviour more broadly has with self-harm. This may give further understanding of binge behaviour and how this may link to other risk behaviours such as self-harm.

The present review included no analyses of moderators. Although small, some heterogeneity was observed between studies which may have been explained by moderator analyses. However, the small number of studies in many of the analyses would have meant moderator analysis would have been underpowered [87]. Likewise, due to small numbers of studies entered into meta-analyses, BED vs. non-clinical controls and BED vs. EDNOS/OSFED could not be assessed for risk of publication bias. Whilst it is important to complete only meaningful funnel plot analyses with ten or more studies [34], the results remain limited by our understanding of publication bias for these meta-analyses. Due to uncertainty when planning the review about what comparisons would be possible given the available literature, no hypotheses were generated, which is a further limitation.

The DSM diagnostic criteria for eating disorders was updated in 2013 [4]. The studies included were published before and after this update. The present review did not look at differences between this time point in diagnostic change, which may have affected results and methods used by the studies. As those experiencing BED are more likely to experience self-harm compared to the non-clinical populations, it is imperative that clinical services regularly screen for self-harm in BED populations and provide effective interventions that target both eating disorder behaviours and self-harm behaviours. As Cognitive Behavioural Therapy (CBT) is recommended in the treatment of BED [88] and for self-harm [19], it is recommended psychotherapies offered to this population include cognitive-behavioural aspects. Further research is needed to understand the efficacy of CBT and other therapies in clinical settings for co-occurring BED and self-harm. Effective interventions may be limited by the lack of recognition and understanding of BED [3, 9] compounded by the scarce research in this area. Issues with the recognition and understanding of BED may mean self-harm risk is also not identified, which increases risk of further distress and suicide [22]. The present review contributes to the existing literature indicating thar BED is as concerning as other eating disorder diagnoses in terms of self-harm. It is hoped this finding contributes to removing stigmas around BED to recognise the great distress associated with this eating disorder.

This systematic review and meta-analysis indicates that lifetime self-harm is more prevalent in people who experience BED compared to non-clinical control groups but does not differ significantly to AN and EDNOS/OSFED groups. Notably self-harm was lower in BED compared to BN. This review highlights a crucial gap in our understanding of those experiencing BED. To better support this population, future research should investigate the causes and patterns of self-harm in BED groups, including the temporal characteristics of this relationship, to inform effective interventions, improve access to services and help reduce stigma experienced by those experiencing BED.

## Electronic Supplementary Material

Below is the link to the electronic supplementary material.

Supplementary Material 1

## Data Availability

All data generated or analysed during this study are available in this published article (and its supplementary information files).

## Abbreviations

BED: Binge Eating Disorder
AN: Anorexia Nervosa
BN: Bulimia Nervosa
OSFED: Other Specified Feeding and Eating Disorders
EDNOS: Eating Disorder Not Otherwise Specified
ARFID: Avoidant and Restrictive Feeding and Intake Disorder
DSM: Diagnostic and Statistical Manual of Mental Disorders
