# Supplementary material for: Self-harm in individuals who experience binge eating disorder: A systematic review and meta-analysis
Source: J Eat Disord. 2025 Sep 2;13:198. doi: 10.1186/s40337-025-01379-8 (PMC12406376; doi:10.1186/s40337-025-01379-8)
Supplement: Supplementary file 1 — Supplementary Material 1 [file 40337_2025_1379_MOESM1_ESM.docx]

**Supplementary File 1**

**Search strategy per database**

| Search block | PsycINFO | MEDLINE | Web of Science^a^ |
| --- | --- | --- | --- |
| Self-harm | self-harm.mp.  OR  self harm.mp.  OR  self-injur*.mp.  OR  self injur*.mp.  OR  self-wound* .mp.  OR  self wound*.mp.  OR  self-mutilat*.mp.  OR  self mutilat*.mp.  OR  self-poison*.mp.  OR  self poison*.mp.  OR  parasuicid*.mp.  OR  non-suicid*.mp.  OR  non suicid*.mp.  OR  NSSI.mp.  OR  DSH.mp. | self-harm.mp.  OR  self harm.mp.  OR  self-injur*.mp.  OR  self injur*.mp.  OR  self-wound*.mp.  OR  self wound*.mp.  OR  self-mutilat*.mp.  OR  self mutilat*.mp.  OR  self-poison*.mp.  OR  self poison*.mp.  OR  parasuicid*.mp.  OR  non-suicid*.mp.  OR  non suicid*.mp.  OR  NSSI.mp.  OR  DSH.mp. | TS=self-harm  OR  TS=self harm  OR  TS=self-injur*  OR  TS=self injur*  OR  TS=self-wound*  OR  TS=self wound*  OR  TS=self-mutilat*  OR  TS=self mutilat*  OR  TS=self-poison*  OR  TS=self poison*  OR  TS=parasuicid*  OR  TS=non-suicid*  OR  TS=non suicid*  OR  TS=NSSI  OR  TS=DSH |
| AND | | | |
| BED | exp Binge Eating/  OR  binge.mp.  OR  exp Binge Eating Disorder  OR  exp Eating Behaviour  OR  Binge disorder.mp.  OR  BED.mp. | exp Binge Eating/  OR  binge.mp.  OR  exp Binge Eating Disorder  OR  exp Eating Behaviour  OR  Binge disorder.mp.  OR  BED.mp. | TS=BINGE  OR  TS=BINGE EAT*  OR  TS=BINGE DISORDER  OR  TI=BED |

^a^ ‘BED’ was searched in titles (TI), rather than across topic searches (TS), due to its high frequency of unrelated search returns.

**Supplementary File II**

**Forest plots**

*Forest plot of BED compared to BN meta-analysis*


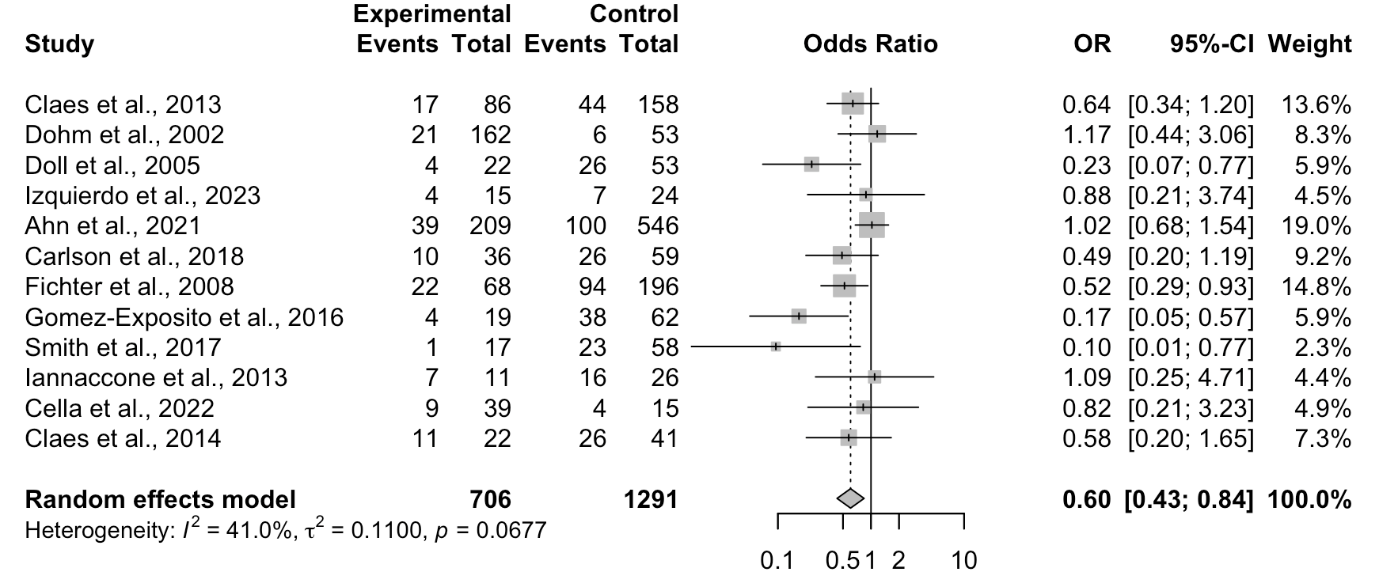


*Forest plot of BED compared to AN meta-analysis*

**
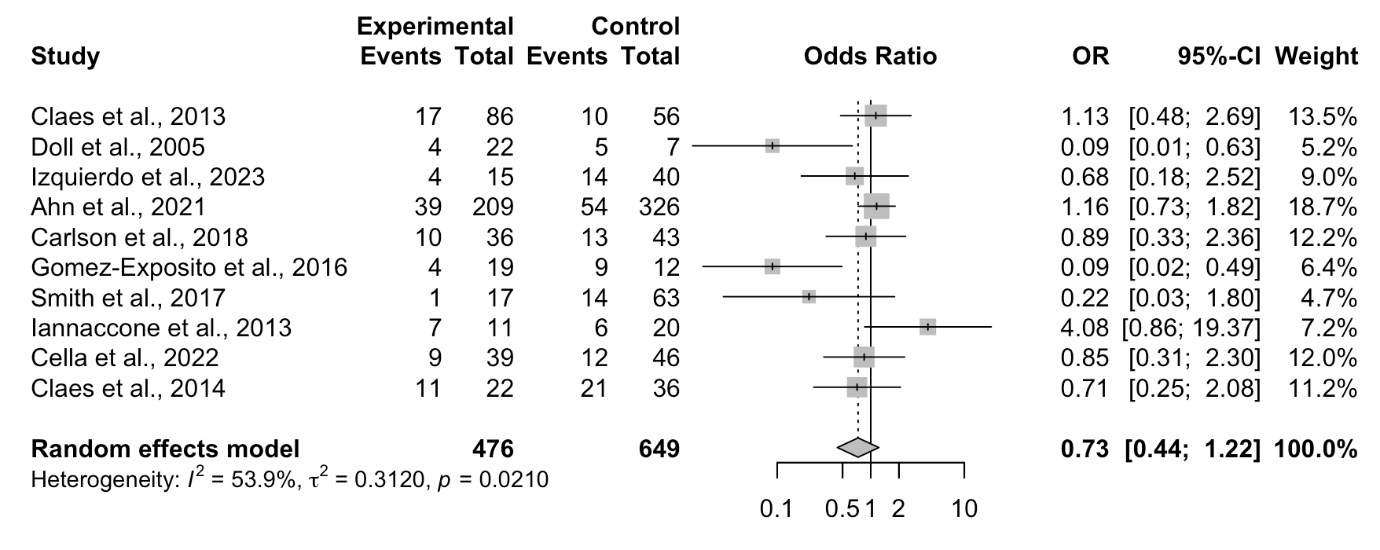
**

*Forest plot of BED compared to EDNOS/OSFED meta-analysis*


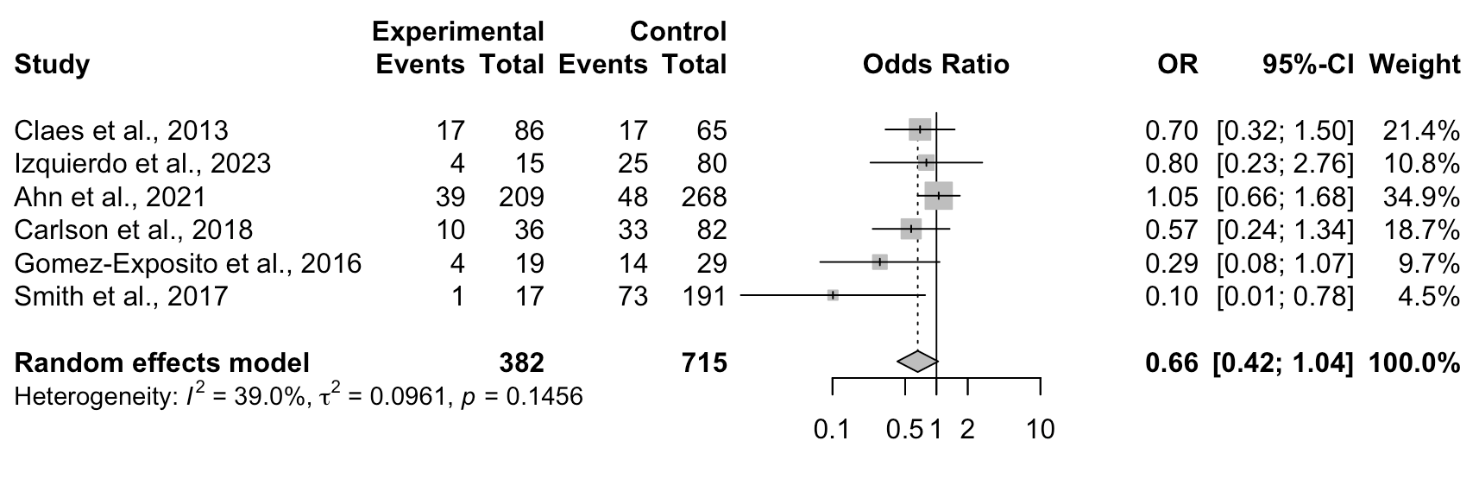


*Forest plot of BED compared to non-clinical controls meta-analysis*
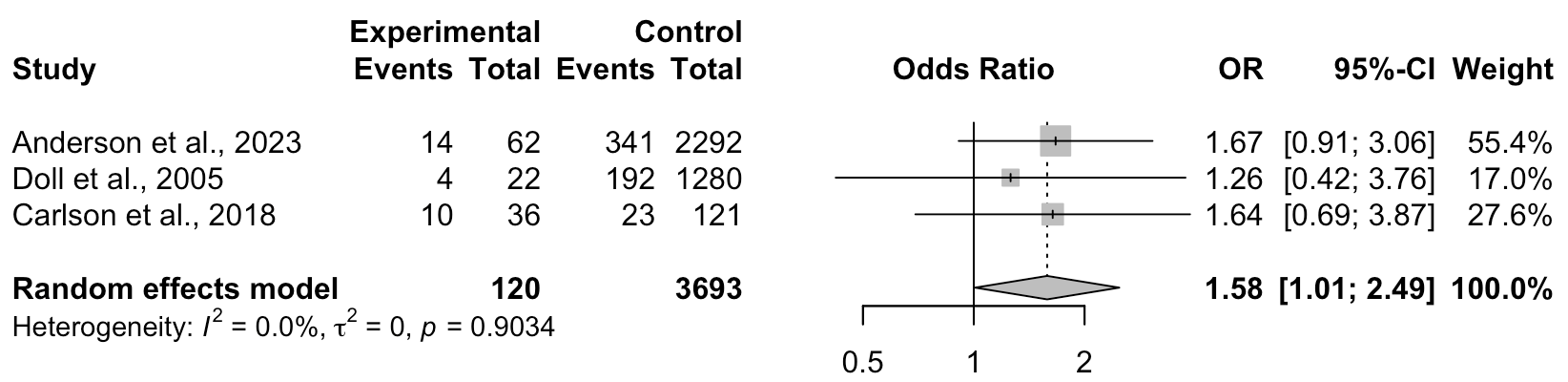


**Supplementary File III**

### Funnel Plots to assess risk of publication bias

*BED vs BN risk of publication bias (funnel plot)*


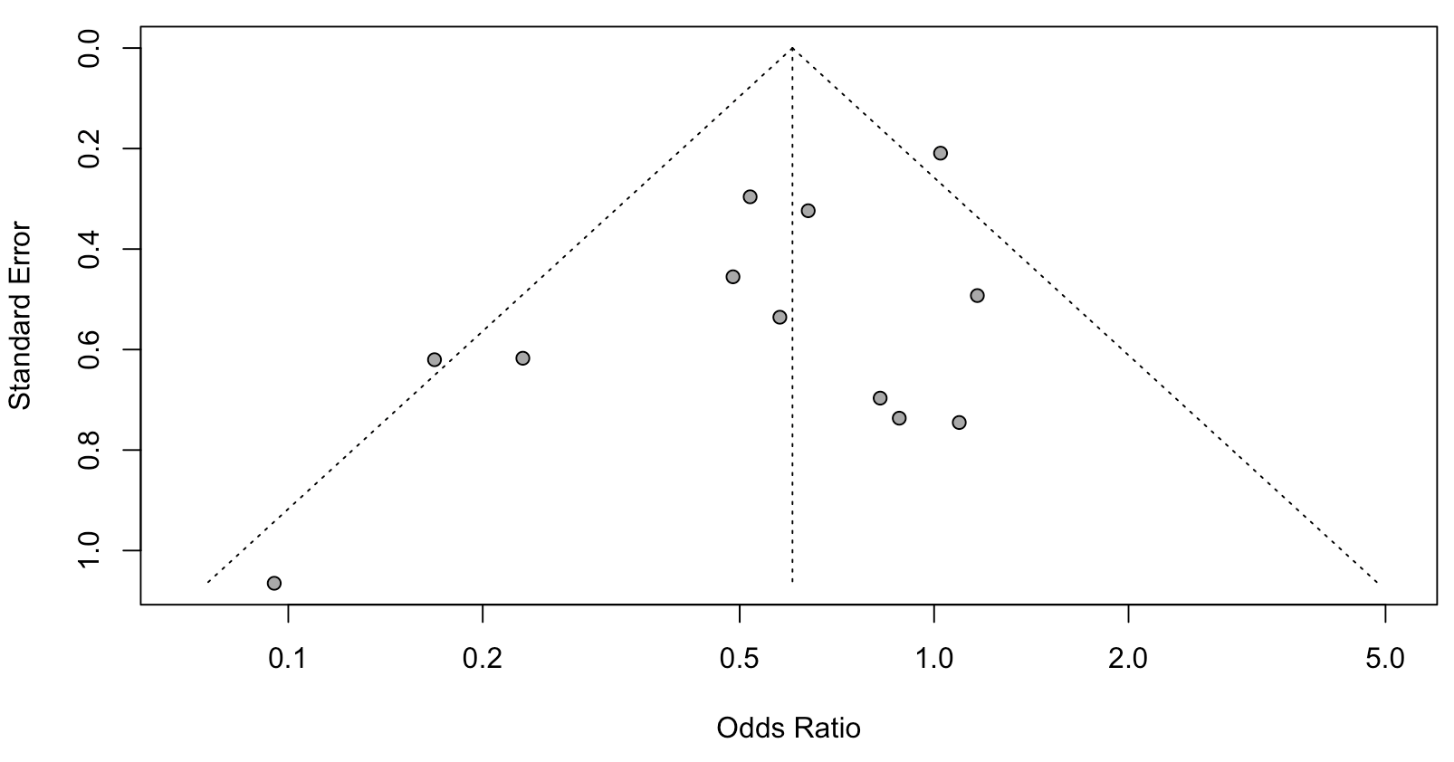


*BED vs AN risk of publication bias (funnel plot)*

**
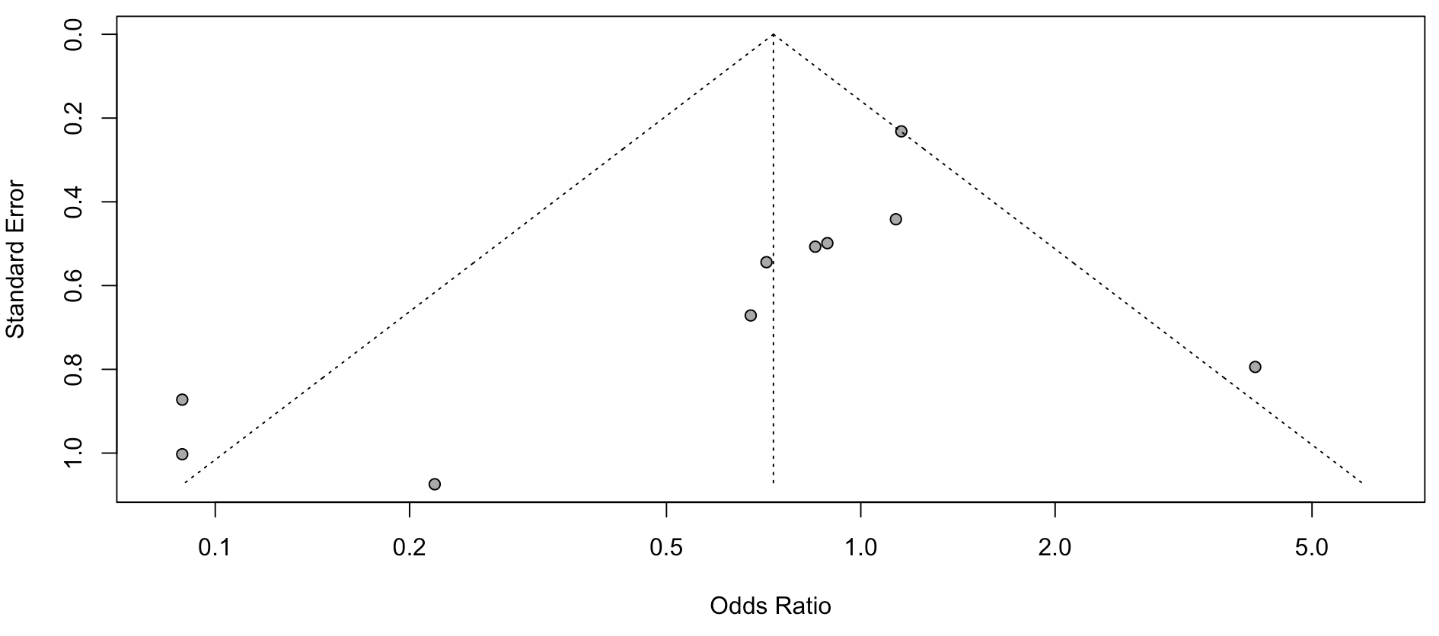
**
